# The Free Energy Profile of Tubulin Straight-Bent Conformational Changes, with Implications for Microtubule Assembly and Drug Discovery

**DOI:** 10.1371/journal.pcbi.1003464

**Published:** 2014-02-06

**Authors:** Lili X. Peng, Monica T. Hsu, Massimiliano Bonomi, David A. Agard, Matthew P. Jacobson

**Affiliations:** 1 Department of Pharmaceutical Chemistry, University of California, San Francisco, San Francisco, California, United States of America; 2 Graduate Group in Biophysics, University of California, San Francisco, San Francisco, California, United States of America; 3 Department of Bioengineering and Therapeutic Sciences, University of California, San Francisco, San Francisco, California, United States of America; 4 Howard Hughes Medical Institute and Department of Biochemistry and Biophysics, University of California, San Francisco, San Francisco, California, United States of America; 5 Department of Biochemistry and Biophysics, University of California, San Francisco, San Francisco, California, United States of America; University of Notre Dame, United States of America

## Abstract

αβ-tubulin dimers need to convert between a ‘bent’ conformation observed for free dimers in solution and a ‘straight’ conformation required for incorporation into the microtubule lattice. Here, we investigate the free energy landscape of αβ-tubulin using molecular dynamics simulations, emphasizing implications for models of assembly, and modulation of the conformational landscape by colchicine, a tubulin-binding drug that inhibits microtubule polymerization. Specifically, we performed molecular dynamics, potential-of-mean force simulations to obtain the free energy profile for unpolymerized GDP-bound tubulin as a function of the ∼12° intradimer rotation differentiating the straight and bent conformers. Our results predict that the unassembled GDP-tubulin heterodimer exists in a continuum of conformations ranging between straight and bent, but, in agreement with existing structural data, suggests that an intermediate bent state has a lower free energy (by ∼1 kcal/mol) and thus dominates in solution. In agreement with predictions of the lattice model of microtubule assembly, lateral binding of two αβ-tubulins strongly shifts the conformational equilibrium towards the straight state, which is then ∼1 kcal/mol lower in free energy than the bent state. Finally, calculations of colchicine binding to a single αβ-tubulin dimer strongly shifts the equilibrium toward the bent states, and disfavors the straight state to the extent that it is no longer thermodynamically populated.

## Author Summary

Microtubules are composed of αβ-tubulins that play an instrumental role in regulating intracellular trafficking and formation of the mitotic spindle during mitosis and cell division. Structural studies have shown that tubulin exists in a “straight” conformation compatible with that in the microtubule lattice and a “bent” conformation thought to represent the unassembled state. There is current debate as to whether the straight-to-bent conformational change in tubulin is the cause or consequence of tubulin's assembly into the microtubule lattice. Here, we use free-energy molecular dynamics simulations to qualitatively understand the conformational landscape of tubulin in the unassembled state and upon lateral binding. We predict that soluble tubulin exists primarily in a bent conformation; our simulation results show that tubulin primarily adopts an intermediately bent conformation in agreement with structural data. We also show that lateral binding of two tubulins shifts the equilibrium in favor of the “straight” state, supporting the hypothesis that the straight-to-bent conformational change is the consequence of tubulin's incorporation into the microtubule lattice via lateral interactions. We also show that colchicine binding shifts the population of tubulin in favor of a bent state, further implicating our work in drug discovery.

## Introduction

Microtubules (MTs) are dynamic cytoskeletal polymers formed by the polymerization of αβ-tubulin, a globular heterodimer comprised of two structurally related 55 kDa α- and β-subunits. Microtubules play a vital role in intracellular trafficking and cell division; these functions are influenced by the complex dynamics of the MT plus end, which undergoes stochastic periods of assembly and disassembly. The role of αβ-tubulin conformational changes in the processes of assembly and disassembly has been the subject of great interest and some controversy [1]. Here we investigate the free energy landscape of αβ-tubulin using molecular dynamics simulations, emphasizing implications for models of assembly, and modulation of the conformational landscape by colchicine, a tubulin-binding drug that inhibits microtubule polymerization.

Tubulin has been shown to exist in two extreme conformations: a “straight” conformation observed in antiparallel zinc-induced tubulin sheets [2]–[6], which is compatible with incorporation into the MT lattice, and a “bent” conformation observed in the structure (T2R complex) of tubulin bound with colchicine, a MT-destabilizing drug, in a complex with the stathmin-like domain of RB3 (SLD-RB3) [7]. (The resolution of this T2R-colchicine structure was recently enhanced to 2.73 Å [8].) X-ray crystallographic studies have also shown similarly bent tubulin structures in a complex with SLD-RB3 [9], bound to other MT-destabilizing drugs (vinblastine [8] and podophyllotoxin), as well as MT-stabilizing drugs (epothilone A and zampanolide) in a complex with tubulin tyrosine ligase (TTL) [10]. Distinguishing the straight and bent tubulin conformations are conformational rearrangements of the intermediate domains and an intra-dimer rotation: rotations of ∼8° and ∼11°, are required to superimpose the intermediate domains of the α- and β-subunits, respectively. In addition a ∼12° intradimer rotation is required to superimpose both subunits of the straight and bent tubulin structures. Finally, the “straight” and bent tubulins are also distinguished by local rearrangements in the M and H1-S2 loop on both subunits, the β-subunit T7 loop and H8 helix, and the α-subunit T5 and H6-H7 loops [4], [7], [11].

**Figure 1:**
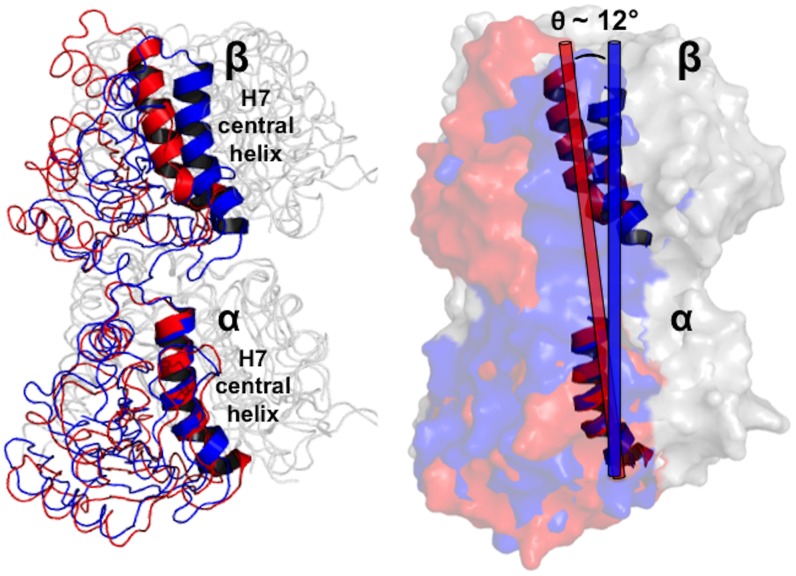
Conformational rearrangements of α- and β-subunits of the straight (PDB entry 1JFF [4], resolution 3.5 Å) and bent (PDB entry 1SA0 [7], resolution 3.58 Å) tubulin heterodimers. The N-terminal nucleotide binding (residues 1–205) and C-terminal (residues 382–437) domains are rendered as grey ribbons. Helices and β-strands comprising the intermediate domain (residues 206–381) are colored blue (straight) and red (bent). We quantify curvature of the straight and bent heterodimers based on a ∼12° intradimer rotation between the α- and β-H7 helices (residues 222–244), after superimposing the α-H7 helices. Figure was generated using PyMOL [49].

Multiple rotational and translational motions in the α- and β-subunits differentiate the “straight” and “bent” conformations. We describe the ∼12° intradimer conformational change of the heterodimer, as described originally by Knossow et al., by the movement of the H7 central helices in the α- and β-subunits (see Figure 1). It is noteworthy that our method of defining the curvature of a tubulin heterodimer differs from that used by Voth and co-workers, who defined the ‘intrinsic bending angle’ by calculating the intersection angle of two least-square fitted-vectors, each defined through the center-of-masses of the N-terminal, intermediate, and C-terminal domains of each subunit [12]–[14]. Voth et al. also characterized rotational motions of tubulin by the twist angle between the α- and β-subunits. In Results and Discussion, we quantitatively compare our method of calculating the intradimer rotation angle with Voth's method of calculating the ‘intrinsic bending angle’.

The role of nucleotide state and tubulin conformation has been debated in the context of two competing models of microtubule assembly [15]–[17]. The allosteric model posits that GTP binding to the exchangeable nucleotide-binding site (E-site) on the β-subunit of soluble tubulin induces a “straight” conformation competent for polymerization. On the other hand, the lattice model posits that conformational changes in unpolymerized tubulin occur upon recruitment into the growing lattice, and the role of GTP is to increase affinity for the lattice by strengthening longitudinal contacts. Both experimental and computational approaches have been utilized to study this straight-to-bent conformational change in tubulin associated with incorporation into the MT lattice. Using small-angle X-ray scattering (SAXS), Rice et al. showed that, under conditions where tubulin does not polymerize, soluble GTP- and GDP-bound tubulin adopt conformations that were indistinguishable based on the SAXS profiles, and consistent with structures of bent tubulin [1]. As aforementioned, the conformational landscape of tubulin has also been examined by molecular dynamics simulations by Voth and co-workers [12]–[14]. In Voth et al.'s unrestrained molecular dynamics simulations of up to 120 ns in length, these authors reported that tubulin explores many conformations including bent structures similar to that observed in the T2R-colchicine complex [14]. This bend direction is in agreement with those reported in Bennett et al.'s 20-ns MD simulations of GTP- and GDP-bound unpolymerized tubulin [18]. These previously reported molecular dynamics simulations also examined differences between GTP- and GDP-bound tubulin, an issue that we do not consider here.

In this study we utilize free-energy calculations to characterize the conformational landscape of a tubulin heterodimer, interpolating between the “straight” structure from Zn^2+^-induced protofilaments and “bent” tubulin from the T2R-colchicine complex. In contrast to unrestrained molecular dynamics simulations, we explore tubulin conformations only along a coordinate connecting these two states, which we quantify using an intradimer rotation angle (Figure 1). However, by performing umbrella sampling and analysis using the weighted histogram analysis method (WHAM), we can estimate the free energy associated with deforming tubulin along this coordinate, providing quantitative predictions concerning the relative free energies of the bent and straight states. Specifically, this “potential of mean force” (PMF) predicts that tubulin can exist in a continuum of conformations ranging between straight and bent, but in agreement with existing structural data, suggests that the bent states have lower free energy and thus dominate in solution.

**Figure 2:**
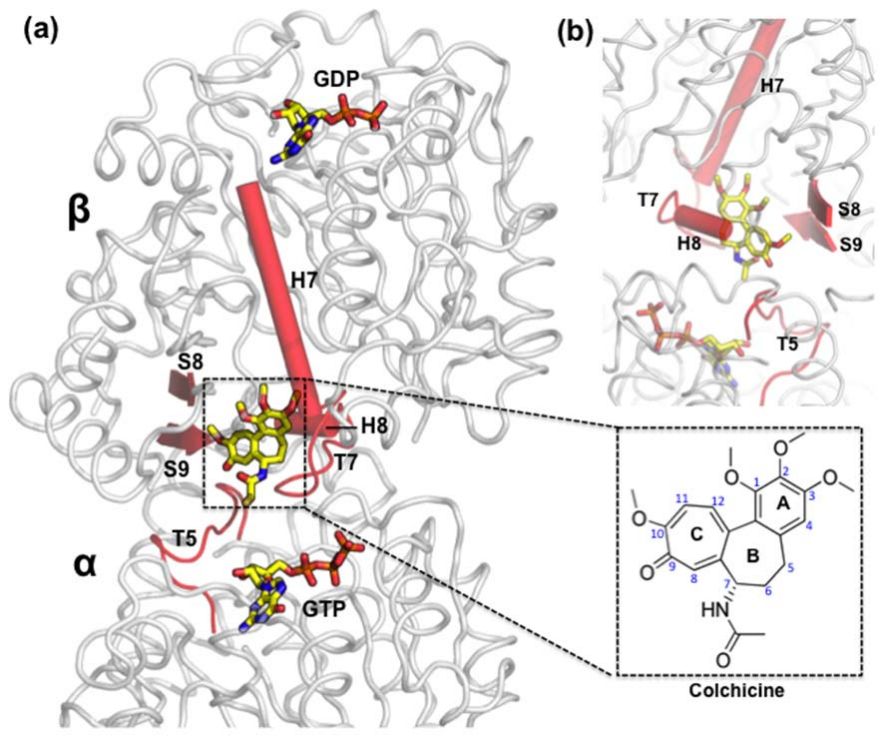
Crystal structure of the colchicine-bound soluble tubulin from the bovine tubulin:RB3-stathmin-like domain (SLD) (T2R) colchicine complex. a) The T2R-colchicine complex is a head-to-tail longitudinal assembly of two αβ-tubulin heterodimers. b) 90° rotated view of the colchicine binding site. Figure was generated using PyMOL.

We also consider how a MT-disrupting drug modulates the equilibrium between the straight and bent tubulin conformations [19]. The oldest microtubule-disrupting drug, colchicine, was discovered in 1889; its action on tubulin was elucidated in 1949 [11]. Crystal structures have revealed that colchicine binds close to the interface between the α- and β-subunits (Figure 2), with the binding site primarily on the β-subunit. Colchicine binds to the soluble, unassembled form of tubulin, forming a poorly reversible colchicine-tubulin complex. The binding site is sterically occluded in the straight conformation, thus colchicine inhibits tubulin polymerization, however the complex can be incorporated into the microtubule lattice at both plus and minus ends [11], [20]. It is thought that colchicine binding also displaces the M loop on the β-subunit (Phe β270 to Val β286), the structural element instrumental in establishing the lateral contacts with the tubulin molecule in the neighboring protofilament, further preventing tubulin from adopting a polymerization-competent structure [7]. Substoichiometic concentrations of the tubulin-colchicine complex are sufficient to inhibit microtubule growth, whereas high TC concentrations lead to microtubule depolymerization [21]. Previous research has argued that colchicine binding strongly favors binding the bent tubulin conformer, colchicine is sterically hindered from binding the straight tubulin structure [22]. Our computational results further support a conformational-bias mode of action for colchicine; the PMF shows that colchicine binding strongly disfavors the straight conformation.

## Methods

### Preparation of endpoint structures of conformational landscapes

#### Selection of straight and bent tubulin endpoint structures

Conformational change coordinates for tubulin are bounded by the taxol-liganded straight tubulin heterodimer, observed in zinc-induced protofilament sheets (PDB id 1JFF, resolution 3.5 Å, organism *B. taurus*), and the bent tubulin structure observed in the α_1_β_1_ heterodimer of the T2R-colchicine complex (PDB id 1SA0, resolution 3.58 Å, organism *B. taurus*) (see Figure S1 in Text S1). For the simulations of laterally-paired tubulins, the endpoints of the conformational change coordinate are terminated by laterally-paired straight and bent tubulins, each with α-α and β-β protofilament contacts, as described in Wells et al [23]. There are several other crystal structures that show tubulin in a bent conformation, including some with better resolution [8], [9], [24]. We consider below the relationship of these structures to the conformational coordinate calculated in this work. We selected 1SA0 to define the bent endpoint because (1) it has the same sequence (bovine) as the selected straight tubulin structure, (2) it has a conformation that is somewhat more ‘bent’ than the other structures, and (3) it includes colchicine, making it particularly appropriate for defining changes in the free energy profile caused by colchicine binding. These endpoint structures were processed as follows.

#### Unpolymerized tubulin

For the straight tubulin endpoint structure, taxol and the Zn^2+^ ion were removed from 1JFF while preserving the Mg^2+^ ion-coordinated GTP molecule on the N-site of the α-subunit and the GDP molecule on the E-site of the β-subunit. Missing residues of the α-H1-S2 loop (residues α:35–60) were incorporated using those in Nogales et al.'s earlier Zn^2+^-induced protofilament model (PDB entry 1TUB [6]). For bent tubulin, colchicine was removed from the α_1_β_1_ heterodimer in 1SA0 while preserving the Mg^2+^ ion-coordinated GTP molecule on the α-subunit N-site and GDP molecule on the β-subunit E-site. The PRIME 3.1 module [25] from the Schrödinger 2012 suite of programs was used to predict loop conformations for the remaining structurally unresolved residues in the α-subunit H1-S2 loop (residues α:35–60) and the β-subunit M loop (β:271–283).

These two modified endpoint structures were then energy-minimized and equilibrated using the GROMACS 4.5.5 package [26]. The GROMOS96 43a1 force field [27] was used in conjunction with the particle mesh Ewald sum method [28] to treat long-range electrostatic interactions. A time step of 2 fs was used for all simulations. Parameters for GTP and GDP were generated from the GlycoBioChem PRODRG2 server [29]. Each endpoint structure was solvated in explicit SPC water molecules^22^ in the center of a periodic cubic box (dimensions of 125 Å×125 Å×125 Å) and neutralized with NaCl. The GROMACS 4.5.5 *g_mindist* module was used to ensure that there was no overlap between the system with its periodic image during equilibration. Each system was energy-minimized using steepest descent while applying Ferguson's flexible SPC water model [30] constraints on the solvent. Periodic boundary conditions were applied for each system throughout all minimization and equilibration steps. Equilibration was performed in two stages: first, the system was heated to 300 K, through use of velocity rescaling, in the isothermal-isotropic (NVT) ensemble while applying position restraints on the entire system. Each system had attained 300 K by 100 ps, at which point the structure was extracted and further equilibrated in the isothermal-isobaric (NPT) ensemble (300 K, 1.013 bar) for up to 500 ps using the Parrinello-Rahman barostat [31] implemented in GROMACS 4.5.5.

#### Laterally-bound tubulin pairs

For the straight pair endpoint, two straight tubulins laterally paired in the α-α, β-β manner were taken from Wells et al.'s cryo-EM-derived “N” model of the MT lattice [23], of which the atomic coordinates were obtained through rigid fitting to Downing et al.'s 8 Å density map of a 13-protofilament MT [32]. To ensure that the interdimer contacts represent those stabilizing α-α, β-β lateral contacts, Wells et al. performed a multi-stage flexible fitting and equilibration procedure on the “N” tubulin pair totaling 85 ns. For the bent pair endpoint, we preserved these interdimer lateral contacts when globally aligning the unpolymerized bent tubulins to the positions of the laterally-bound straight tubulins. Both laterally-bound tubulin structures were solvated with explicit SPC water molecules in the center of a periodic box (dimensions of 300 Å×300 Å×300 Å). Energy minimization and equilibration procedures were performed as for the unpolymerized tubulin described previously; the duration of the NVT and NPT equilibration phases were 1 ns and 3 ns, respectively.

#### Colchicine-bound tubulin

The α_1_β_1_ heterodimer from the T2R-colchicine complex was used as the bent endpoint, preserving the colchicine molecule. Solvation, minimization, and equilibration of this structure were performed in analogous fashion as for the unpolymerized bent tubulin. The GlycoBioChem PRODRG2 server [29] was used to generate parameters for colchicine. To create a (hypothetical) structure of colchicine-bound straight tubulin, we used a stepwise procedure involving successive iterations of global alignment of tubulin and superimposition of colchicine for tubulin structures intermediate between the bent to straight endpoints, as described in greater detail in “Generating the morphing path”.

### Selecting the reaction path

We chose the path collective variable, or reaction path, to be the angle required for superimposing the secondary structural elements of the α- and β-subunit intermediate domains (helices H6, H7, H8, H9, H10 and strands S7, S8, S9, and S10) of the straight and bent tubulin molecules. The conformational space *R* relative to this path can be expressed in terms of two collective variables, *s*(*R*), which represents the progress of the dynamics along the conformational change coordinate, and *z*(*R*), which represents the progress away from the reaction path, as follows [33]–[36]:
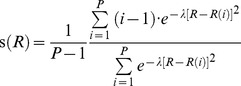
(1)

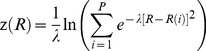
(2)where *i* is a discrete index ranging from 1 to *P*, the total number of structures along the reaction coordinate, 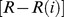 is the mean squared displacement of the 167 Cα atoms of the α- and β-intermediate domain secondary structural elements, and *λ* is a prefactor in the exponential term that defines both *s*(*R*) and *z*(*R*). (For the laterally-paired tubulins, 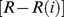 includes the two sets of 167 Cα atoms in both dimers.) The value of *λ* is chosen to be proportional to the inverse of the mean square displacement between two successive frames along the reaction coordinate; here we set 
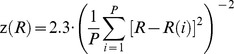
. It is important to emphasize that umbrella sampling along this defined reaction coordinate only allows us to explore the free energy differences for tubulin conformations linearly interpolated along the defined reaction path; this method does not allow us to explore bent tubulin states whose α- and β-subunit intermediate domain rotation deviates substantially from the defined reaction path.

### Generating the morphing path

#### Unpolymerized tubulin and laterally-paired tubulins

The thermally equilibrated straight and bent tubulin endpoint structures were submitted to the Molmov morphing server [37] to generate thirteen equidistantly-spaced, energy-minimized intermediate *apo* tubulin structures along a linear path. These thirteen *apo* structures were then liganded with GTP, GDP, and the Mg^2+^ ion as follows: 1) each of the two endpoint structures was globally aligned with its nearest *apo* intermediate; 2) GTP, GDP, and the Mg^2+^ ion were superimposed onto the nearest intermediate structure, which was then solvated, minimized, and equilibrated using the methods described in the previous section. This cycle was iterated from both endpoints for each successive tubulin intermediate. The final snapshots of the fifteen equilibrated tubulin configurations (straight, fully bent, and thirteen intermediately bent structures) were used as the initial *P = *15 umbrella nodes along the reaction path for the calculating the potential of mean force for unpolymerized tubulin (Figure S2a in Text S1). This procedure was executed in an analogous fashion to produce the final snapshots for the thirteen equilibrated laterally-bound tubulin pair configurations (straight pair, fully bent pair, eleven intermediately bent pairs) for the initial *P = *13 umbrella nodes in subsequent potential of mean force calculations (Figure S2c in Text S1).

#### Colchicine-bound tubulin

The absence of a colchicine-bound straight tubulin structure required us to use the equilibrated straight endpoint structure, along with the equilibrated colchicine-bound bent endpoint structure, to generate the colchicine-bound tubulin intermediates. These two structures were submitted to Molmov [37] to produce seven equidistantly-spaced, energy-minimized intermediate *apo* tubulin structures. These seven *apo* structures were complexed with colchicine, GTP, GDP, and the Mg^2+^ ion as follows: 1) the equilibrated colchicine-bound fully bent tubulin complex was globally aligned with its nearest *apo* intermediate; 2) colchicine, GTP, GDP, and Mg^2+^ from the equilibrated colchicine-bound fully bent tubulin were then superimposed onto the nearest *apo* intermediate; and 3) solvation, minimization, and equilibration were performed on the successive *holo* tubulin intermediate as described above. This cycle was repeated for each successive intermediate from the bent to straight endpoints until the colchicine-bound straight tubulin had been fully equilibrated. The final snapshots for the nine equilibrated colchicine-bound tubulin configurations (straight, fully bent, and seven partially bent intermediates) were used as the initial *P = *9 umbrella nodes along the reaction coordinate for calculating the potential of mean force for colchicine-bound tubulin (Figure S2b in Text S1).

### Umbrella sampling, potential of mean force simulations

Harmonic biasing potentials are placed at each umbrella position along a collective variable. The harmonic biasing potentials for *s*(*R*) and *z*(*R*) are expressed as:
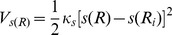
(3)

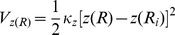
(4)where 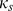 and 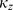 are the force constants for the harmonic restraints for *s* and *z*, and *R*_i_ is each umbrella position *i* within the configurational space *R*.

For the unpolymerized tubulin, we initially defined *P = *15 equidistantly-spaced umbrella windows along the *s*(*R*) coordinate. However, this discretization proved later insufficient to characterize the free energy along *s*(*R*), so we increased the number of umbrella windows to *P = *35 while restraining each umbrella node by 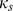 = 10 kcal/mol. To sufficiently characterize the free energy landscape for the laterally-bound tubulin pair, we increased the initial *P = *13 equidistantly-spaced umbrella windows to *P = *50 (Figure S3b in Text S1) while applying restraints of 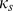 = 10 kcal/mol. Because the domain rearrangements characterizing the straight-to-bent conformational change are fully captured by the *s*(*R*) collective variable, we did not place any harmonic restraints on the *z(R)* path for the unpolymerized tubulin and laterally-paired tubulin dimers.

We used the initial nine colchicine-bound tubulin morphing intermediates to define *P = *9 umbrella windows along *s*(*R*). To achieve adequate sampling among successive umbrella windows along *s*(*R*), we optimized 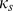 to 10 kcal/mol for a total number of *P = *31 nodes. To prevent colchicine unbinding, especially from the conformations with lower intradimer curvature (0°≤θ≤6°), we restrained the dynamics along the *z*(*R*) coordinate to 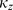 = 10 kcal/mol.

All simulations for the unpolymerized tubulin, laterally-paired tubulins, and colchicine-bound tubulin were performed in the canonical (NVT) ensemble at 1.013 bar and 300 K for 4 ns using the PLUMED 1.3 [38]-implemented version of GROMACS 4.5.5. The last 3 ns of the production runs were used for calculating the free energy profiles.

### Generating free energy profiles using WHAM

**Figure 3:**
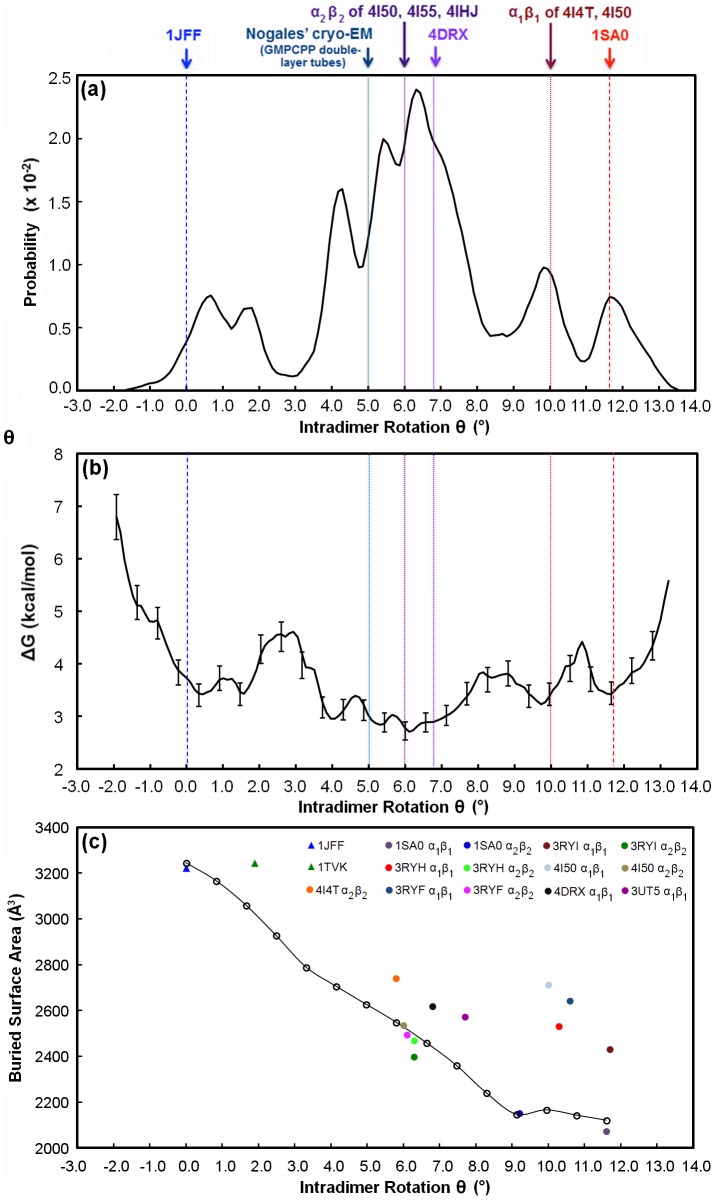
The (a) probability distribution, (b) free energy profile, and (c) buried surface area of unpolymerized tubulin as a function of intradimer curvature. Intradimer rotation of the endpoint structures, the straight taxol-liganded, zinc-stabilized protofilament tubulin and bent α_1_β_1_ heterodimer from the T2R-colchicine complex, are denoted at θ = 0° *(blue dash)* and θ∼12° *(red dash)*, respectively. Intradimer rotation of selected intermediately bent tubulins are denoted as dotted lines: θ∼5° (GMPCPP-tubulin in double-tube layers), θ∼6° (α_2_β_2_ heterodimer of the T2R-TTL-ADP, T2R-TTL-zampanolide, and T2R-TTL complexes), θ∼6.9° (both α_1_β_1_ and α_2_β_2_ heterodimers of the GTP-tubulin-D1 DARPin structure), and θ∼10° (α_1_β_1_ heterodimer of T2R-TTL-zampanolide and T2R-TTL epothilone A complexes). Buried surface area values of tubulin heterodimer structures with resolutions up to 3 Å are shown in panel (c); the structures from PMF in (b) *(open circles)* and crystal structures of the straight *(solid triangle)* and bent *(solid circles)* heterodimers are denoted for each tubulin complex.

**Figure 4:**
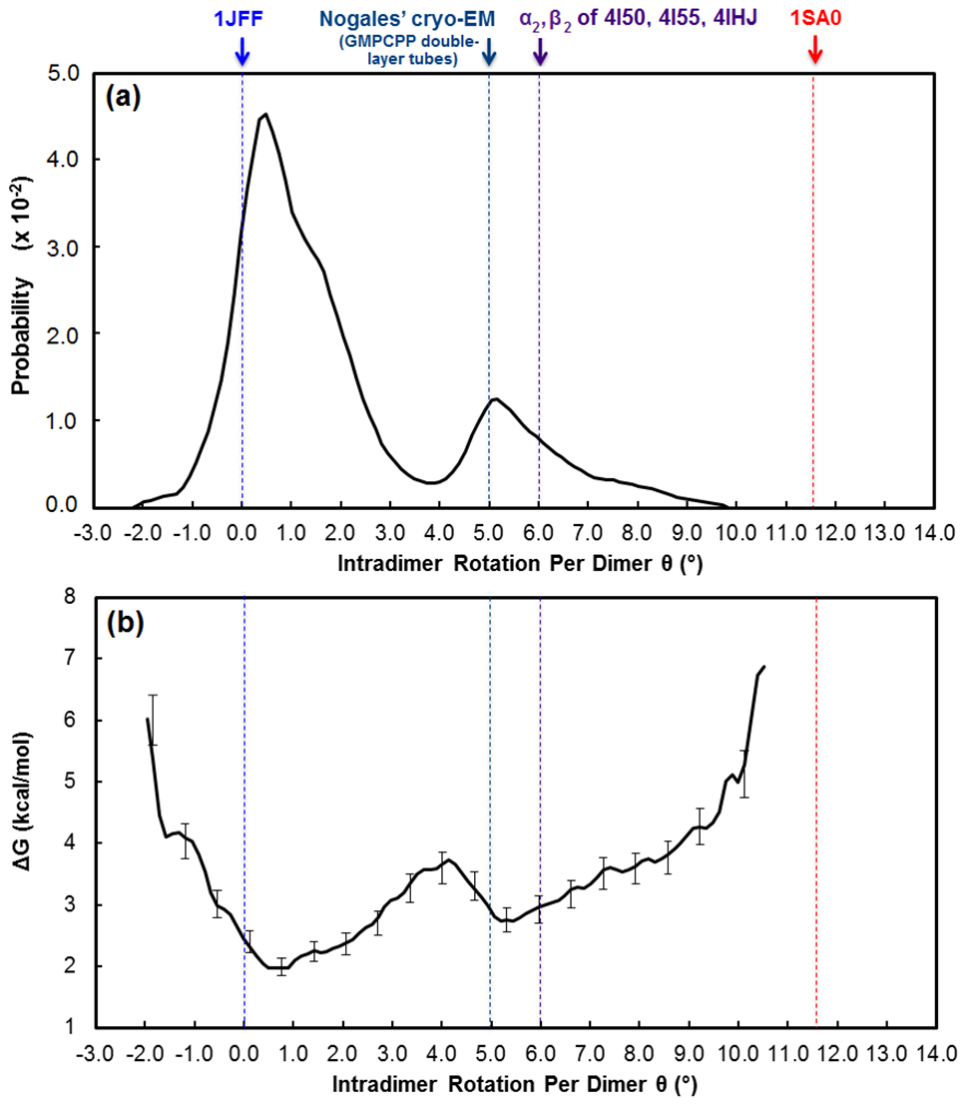
The (a) probability distribution and (b) free energy profile for the laterally-paired tubulins. Probability histograms corresponding to each umbrella window are shown in Figure S2c in Text S1.

The weighted histogram analysis (WHAM) [39], [40] approach was used to merge the data from the molecular dynamics simulations and unbias the umbrella histograms in *s*(*R*). For the unpolymerized tubulin and laterally-paired tubulins, we used the *g_wham* module [41] of GROMACS 4.5.5 to calculate the free energy profiles along the *s*(*R*) path, based on Eqn. 1, using a resolution of 1000 bins and tolerance of 1e-6. We then estimated the statistical uncertainty 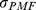 in the *s*(*R*) path accordingly [42]:
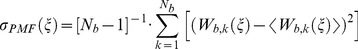
(5)where ξ is the reaction coordinate, 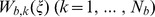 represents each of the *N*_b_ bootstrapped free energy profiles, and 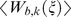 is the average of the *N*_b_ bootstrapped free energy profiles. For each umbrella position ξ_i_, we generated a new bootstrapped trajectory ξ_b,i_(*t*) yielding a new histogram *h*_b,i_(ξ). WHAM was then executed on this new set of histograms for *N*_b_ iterations to compute a bootstrapped free energy profile *W*_b,k_(ξ). (The *g_wham* module generates and aligns 1000 bootstrapped free energy profiles *W*_b,k_(ξ) at the initial position, ξ_i_ = 0, in *s*(*R*) so that the uncertainty at ξ_i_ = 0 is zero.) The uncertainty values along the *s*(*R*) coordinate are reported for the unpolymerized tubulin and laterally-bound tubulin pair in Figures 3b and 4b, respectively.

For colchicine-bound tubulin, we calculated the two-dimensional probability distribution and free-energy surface using the *wham-2d* program (Figures S3a–b in Text S1). Results show that sampling in the *z*(*R*) space remained quite close to the *s*(*R*) path, even at *s*(*R*) positions corresponding to lower tubulin curvature, confirming that the harmonic restraining potential 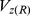 was sufficient to limit considerable deviations from the *s*(*R*) path. Using GROMACS *g_wham*, we then projected this 2D free energy profile onto *s*(*R*) at *z*(*R*) = 0, using 1000 bins and a tolerance of 0.001, and computed the statistical uncertainty 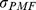 in the *s*(*R*) path using the same bootstrapping methods described above.

### Structural analysis of tubulin conformations

#### Analyzing intradimer curvature of tubulin heterodimers

All calculations of the intradimer rotation angles of each bent tubulin heterodimer with respect to the straight were performed using UCSF Chimera version 1.7 [43]. The Kabsch and Sander algorithm [44] implemented in UCSF Chimera was invoked to assign secondary structures for PDB structures that lacked secondary structure assignments. Each bent heterodimer was structurally superimposed onto the straight taxol-bound, zinc-induced protofilament tubulin by the α-subunit H7 helices. The structural alignment was performed in 3 different ways, using either all atoms, only backbone atoms, or only Cα backbone atoms of the α-H7 helix (see Supplemental Table S3 in Text S1 for exact residues). The intradimer rotation was then defined by fitting a plane to the α- and β-subunit H7 helices using least-squares fitting. The intersection angle of the two vector planes represents the intradimer rotation of each bent heterodimer.

To compute buried surface area, the crystal structures were first pre-processed so that each heterodimer is only complexed with GDP and GTP-Mg^2+^; any other protein chains (i.e. RB3-SLD, TTL, D1), solvent molecules, and counter ions other than Mg^2+^ were also removed. The solvent-accessible surface area of each tubulin heterodimer (*SASA_αβ_*) and the solvent-accessible surface area of each α- and β-subunit, *SASA_α_* and *SASA_β_*, excluding the other subunit, were calculated in PyMOL using a solvent probe radius of 1.2 Å and a solvent density of 4. The buried surface area of each tubulin heterodimer was determined accordingly: *BSA = *(*SASA_α_*+*SASA_β_*)−*SASA_αβ_*.

#### Calculating volume of colchicine binding pocket

For each of the 31 umbrella positions along the conformational change coordinate, five colchicine-bound tubulin structures corresponding to the peak of each histogram were extracted from the last 3 ns of the restrained molecular dynamics trajectories. The SiteMap module [45] in the Schrödinger 2012 suite of programs was used to calculate the volume of the colchicine binding pocket, using the OPLS 2005 force field at a 0.7-Å grid resolution and setting a 6 Å buffer region surrounding colchicine. The SiteScore for each colchicine-tubulin complex was confirmed to be greater than 1. For each of the 31 umbrella positions, the arithmetic mean and standard deviation of the colchicine volume 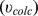 and binding pocket volume 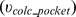 of the five representative structures were calculated.

We also analyzed the hydrogen bonding, hydrophobic interactions, and steric clashes of the colchicine-tubulin complexes at all 31 umbrella positions. For each of the five representative structures from each umbrella position, we used the Maestro command-line “Display Hydrophobic Interactions” script (to identify the atoms of colchicine and tubulin involved in hydrogen bonding and/or hydrophobic interactions. Steric clashes are defined as two atoms separated by a distance that is less than 75% of the sum of their van der Waals radii.

## Results/Discussion

### Unpolymerized tubulin primarily adopts an intermediately bent conformation

#### Free energy profile of unpolymerized tubulin

Figure 3 shows the probability distribution, free energy profile, and buried surface area computed from the potential-of-mean force calculations for unpolymerized tubulin (i.e., a single αβ-heterodimer). We had hypothesized that the PMF would show two prominent basins, reflecting the straight and bent conformations of tubulin. This hypothesis was only partly confirmed. The PMF does show a free energy basin corresponding to the straight conformation, as defined by 1JFF. This basin is separated by a relatively small free energy barrier at ∼3° intradimer rotation from a broad basin that includes, at one end, the bent conformation corresponding to the colchicine-bound structure (1SA0). Integrating the populations, the straight basin (θ<3.5°) accounts for approximately 20% and the various bent states approximately 80%. Although the 1SA0 structure has an intra-dimer rotation that corresponds rather precisely with a local minimum in the PMF, the global minimum in the PMF has an intradimer rotation of ∼6°. However, we note that the free energy difference between this ‘intermediately bent’ global minimum and the ‘fully bent’ minimum at ∼12° is small, <1 kcal/mol, and only slightly larger than the estimates of the statistical uncertainties (the true errors, which would include limitations of the force field, are of course larger). Thus, we cannot confidently conclude, on the basis of these calculations alone, that the intermediately bent state is lower in free energy than the fully bent state.

However, the broad and relatively featureless basin representing the bent states is in itself a striking prediction, suggesting that tubulin can exist in a nearly continuous range of different bent intradimer rotation angles. Soluble GDP-tubulin was previously thought to exist in only two states, the “straight” and “bent” structures represented by PDB ids 1JFF and 1SA0, respectively. Our calculated PMF results are striking because our PMF suggests a broad range of intermediately bent soluble GDP-tubulins whose curvature correspond to specific energy wells in the PMF; this relatively flat landscape is unexpected within the framework of this two-state model. The number of crystal structures of tubulin has been increasing rapidly in the past few years, and many of the newer structures in fact have intradimer rotation angles, when measured in the same manner as for the PMF, that correspond to our ‘intermediately bent’ states (see Table 1). For example, the cryo-EM structures of tubulin observed in double-layer tubes by Nogales et al. [46], several structures of tubulin in complex with tyrosine tubulin ligase, and a GTP-tubulin-D1 DARPin complex [9] all have intradimer rotation angles in the range of 5–7°, similar to the intermediately bent global free energy minimum observed computationally. The combination of the potential of mean force and the comprehensive analysis of crystal structures suggests that tubulin can adopt a wide range of different ‘bent’ states, and we hypothesize, but cannot currently prove, that tubulin in solution is likely to exist in an ensemble of different states, dominated by a variety of bent states. This hypothesis is also supported by other molecular dynamics studies by Voth and co-workers [12]–[14], who observed tubulin interconverting between different intradimer rotation angles on the timescale of tens of nanoseconds.

**Table 1:** Intradimer curvature of “straight” and “bent” tubulin heterodimers.

Structure	Resolution (Å)	PDB ID	Source Organism	Nucleotide State	Hetero dimer	Intradimer Rotation (°)	α-subunit Rotation (°)	β-subunit Rotation (°)	Space Group	Ref.
**“STRAIGHT”**										
Epothilone A-tubulin*	2.89	1TVK	*B. taurus*	GDP	αβ	1.9±0.1	2.8±1.7	1.5±0.6	P21	[5]
Docetaxel-tubulin*	3.70	1TUB	*S. scrofa*	GDP	αβ	4.8±3.3	2.3±0.4	1.9±0.3	P21	[6]
**“BENT”**										
T2R-TTL-zampalonide	1.80	4I4T	*B. taurus*	GDP	α_1_β_1_	10.5±2.7	8.7±1.1	13.3±0.9	P212121	[10]
					α_2_β_2_	5.8±0.2	4.9±1.3	13.3±0.9		
T2R-TTL-ADP	2.00	4IHJ	*B. taurus*	GDP	α_1_β_1_	9.7±1.5	8.7±0.8	14.6±0.9	P212121	[50]
					α_2_β_2_	6.1±0.4	4.3±1.4	12.2±1.0		
T2R-TTL	2.20	4I55	*B. taurus*	GDP	α_1_β_1_	9.7±2.1	8.7±0.9	12.2±4.1	P212121	[10]
					α_2_β_2_	6.1±0.2	6.2±1.2	12.3±0.9		
T2R	2.20	3RYC	*O. aries*	GDP	α_1_β_1_	6.9±0.4	9.8±3.5	10.4±2.1	P212121	[24]
					α_2_β_2_	6.2±0.5	4.2±0.3	11.0±0.5		
GTP-tubulin-D1 DARPin	2.22	4DRX	*O. aries*	GTP	α_1_β_1_	6.8±0.3	11.3±0.4	11.9±1.3	P21	[9]
					α_2_β_2_	6.8±0.3	10.8±0.6	11.9±1.2		
T2R-TTL-epothilone A	2.30	4I50	*B. taurus*	GDP	α_1_β_1_	10.0±2.4	8.5±1.0	13.8±0.9	P212121	[10]
					α_2_β_2_	6.1±0.4	6.0±1.1	12.2±0.7		
T2R	2.40	3RYI	*O. aries*	GDP	α_1_β_1_	11.7±0.6	8.6±0.1	10.8±0.5	P212121	[24]
					α_2_β_2_	6.3±0.3	2.9±0.3	11.0±0.5		
T2R	2.52	3RYF	*O. aries*	GTP	α_1_β_1_	10.6±0.1	7.8±0.6	11.1±0.5	P212121	[24]
					α_2_β_2_	6.1±0.1	4.3±0.1	11.0±0.5		
T2R-colchicine-ustiloxin	2.73	3UT5	*O. aries*	GDP	α_1_β_1_	7.7±0.6	9.8±0.4	10.3±0.3	P212121	[8]
					α_2_β_2_	7.7±0.1	2.5±0.4	11.1±1.0		
T2R	2.80	3RYH	*O. aries*	GMPCPP	α_1_β_1_	10.3±0.2	7.5±0.3	10.8±0.5	P212121	[24]
					α_2_β_2_	6.3±0.6	3.6±0.2	11.1±0.5		
T2R-vinblastine	3.47	4EB6	*O. aries*	GDP	α_1_β_1_	20.4±2.8**	10.2±0.1	10.6±0.5	P212121	[8]
					α_2_β_2_	31.7±0.5**	4.5±0.1	12.3±0.9		
T2R-colchicine	3.58	1SA0	*B. taurus*	GDP	α_1_β_1_	12±0.1	9.2±0.7	11.7±1.6	P65	[7]
					α_2_β_2_	9.2±1.0	7.1±1.1	11.7±1.6		
T2R	3.65	3HKB	*O. aries*	GDP	α_1_β_1_	7.7±1.8	7.6±1.9	13.0±1.8	P65	[20]
					α_2_β_2_	16.5±0.3	7.5±0.4	13.0±1.8		
T2R-podophyllotoxin	4.20	1SA1	*B. taurus*	GDP	α_1_β_1_	12.1±0.6	9.1±0.8	12.9±1.3	P65	[7]
					α_2_β_2_	4.8±0.1	6.8±1.5	13.1±1.0		

For the RB3-SLD-bound structures, the orientations of the longitudinally-paired α_1_β_1_ and α_2_β_2_ heterodimers with respect to the MT plus (+) and minus (−) ends are shown in Figure S4 of Text S1 along with the relative orientations of the D1-(α_1_β_1_) and D1-(α_2_β_2_) dimers in the GTP-tubulin-D1 DARPin structures. Crystal structures are listed in order of decreasing resolution. Residues comprising the α- and β-H7 helices used for angle calculations are listed in Table S3 of Text S1. Each angle was measured using the backbone N-Cα-C-O, Cα, and all atoms, with the standard deviation shown accordingly.

* Control calculations of the “straight” tubulin were performed on the epothilone A- and docetaxel-bound, Zn^2+^-stabilized protofilament tubulin with respect to the taxol-bound, Zn^2+^-stabilized protofilament tubulin (α-H7: 224–242, β-H7: 224–243).

** Outlying values of intradimer tubulin curvature T_2_R-vinblastine structure are likely due to unraveling of the H7 helix.

As aforementioned, our metric for calculating tubulin's curvature focuses on the orientations of the α- and β-H7 helices. Voth et al. accounted for tubulin's curvature by the movement of the α- and β-subunits with respect to one another. To reconcile these two different calculation methods, we utilized Voth's method to measure the intrinsic bending angle of 15 equidistantly-spaced structures along the reaction coordinate for the free energy landscape of unpolymerized tubulin in Figure 3b. The results, shown in Figure S5 in Text S1, indicate that the two metrics, while not identical, correlate strongly with a slope close to 1.

#### Buried surface area of unpolymerized tubulin

It is noteworthy that our method of describing conformational change of tubulin is limited to the rotation of the heterodimer. Voth et al. also calculated a twist angle to account for the twisting motions of the α- and β-subunits with respect to one another, our reaction coordinate does not account for this rotational motion. We recognize limitations in this geometric descriptor, and have also computed the buried surface area (BSA) of various intermediately bent tubulin structures, as an alternative way of quantifying differences between “straight” and “bent” structures (see Figure 3c). These BSA values in Figure 3c track very closely to the conventionally defined intradimer rotation angles of our predicted intermediately bent tubulin structures. Specifically, the interface between the alpha and beta subunits becomes much more extensive as tubulin adopts the straight conformation consistent with incorporation into the microtubule. This suggests a balance of forces between the energy required to straighten the bent dimer and the interfacial packing energy gained in the process. Along the bending coordinate used in our potential of mean force calculations, the buried surface area varies nearly linearly with respect to the geometrical parameter we have used to quantify the intradimer rotation, as might be expected if our reaction coordinate followed a minimum energy path along the landscape. Most of the tubulin structures with resolutions better than 3.0 Å lie close to this line, with the exception of a few structures in highly bent states. It should be emphasized that we have only characterized the free energy landscape along a single coordinate, and other relevant conformational states may exist “off pathway”. Nonetheless, the fact that the crystal structures that we characterized as intermediately bent based on the geometric intradimer angle also have intermediate values of intradimer buried surface area supports the contention that tubulin can adopt an intermediately bent state, and a two-state bent/straight model is thus in certain respects overly simplistic. It is noteworthy that the various X-ray crystallographic structures represented in Figure 3c differ in their space group and resolution.

The α- and β-subunits of our bent endpoint structure have intramonomer rotations of ∼8° and ∼11°, respectively. To examine whether the rotation of the individual the α- and β-subunits is related to the rotation of the entire heterodimer, we calculated the rotation of each α- and β-subunit of the X-ray crystallographic structures of tubulin shown in Table 1, as well as for each tubulin intermediate along the reaction coordinate in Figure 3. (These calculations were performed in accordance with methods described in Knossow et al. [7].) As shown in Table S1 of Text S1, the intramonomer rotations of the α- and β-subunits of the interpolated tubulin structures increase monotonically but nonlinearly with increasing heterodimer curvature. In the X-ray crystallographic structures of bent tubulins, the β-subunit typically rotates between ∼11–14°, whereas the α-subunit intramonomer rotation varies more widely between ∼4–12°. Thus, while our PMFs do not independently explore the intramonomer and the intradimer rotations, the structural information suggests that the intramonomer rotations in the α- and β-subunits may be partially independent degrees of freedom.

Having characterized the surprisingly ‘flat’ free energy profile for tubulin along a bending coordinate, we next examined how this potential of mean force is modulated by (1) lateral association of two tubulin heterodimers (with implications for the lattice model of microtubule assembly), and (2) binding a small molecule drug, colchicine (with implications for microtubule-binding therapeutics).

### Lateral association of tubulin heterodimers favors the straight state

Figure 4 shows the free energy profile and probability distribution for the laterally-paired tubulins. The region of the conformational change coordinate ranging from θ = 10° to θ = 12° was not successfully sampled because the lateral interdimer contacts were not fully preserved during the NPT equilibration of the lateral dimers corresponding to this range of curvature, despite significant effort. These results suggest that unpolymerized tubulin with >10° intradimer rotation is physically incapable of forming both α-α and β-β lateral interactions, unless it transitions into a lower curvature state.

The 1SA0 α_1_β_1_ heterodimer from the T2R-colchicine:tubulin complex exhibits a ∼8.3° twist angle, which is somewhat large compared to the degree of twisting exhibited by the newer X-ray crystallographic structures of tubulins, whose twist angles range from ∼5–7° (see Tables S2a–b in Text S1). We calculated the twist angles of both heterodimers of thirteen laterally-paired tubulin structures and found that, in general, they vary from 2–5°, which are intermediate between the twist angles of the newer crystal structures of bent tubulin, and the twist angle in the straight (taxol-bound) structure. Therefore, because our structures of laterally-paired tubulins along the conformational change coordinate do not exhibit the extreme twisting motion prevalent in the 1SA0 α_1_β_1_ heterodimer, the lateral contacts in our interpolated intermediate structures of laterally-paired tubulins should be stable.

As aforementioned, the role of nucleotide state in affecting tubulin conformation has been intensely debated in the context of the lattice and allosteric models of microtubule assembly. The lattice model postulates that soluble tubulin undergoes conformational changes only upon incorporation into the microtubule lattice and independently of nucleotide state, whereas the allosteric model posits that GTP binding pre-structures soluble tubulin into the “straight” conformation compatible with that in the MT lattice. Our PMF of the laterally-paired tubulins suggests that lateral binding of tubulin heterodimers substantially modifies the free energy profile, consistent with the lattice model. Consistent with our expectations, based on the lattice model, lateral binding strongly shifts the population toward the straight conformation (∼75%), which again is separated from the intermediately bent conformation by a small free energy barrier (∼2 kcal/mol), in this case at ∼4°. The global free energy minimum along the PMF has an intradimer rotation angle (0.5°) very close to that observed in structures of zinc-induced protofilament sheets (1JFF). Although we find it geometrically infeasible for both tubulins to be in a fully bent state, both can exist in an intermediately bent state when laterally associated, and this state represents ∼25% of the population. (The two tubulins are constrained to maintain the same intradimer angle across the PMF, i.e., we have not investigated laterally associated straight-bent pairs, although geometrically we expect these to be unfavorable.) Overall, comparison of the ‘free’ and laterally-paired tubulin PMFs suggests that lateral association shifts the bending equilibrium by ∼1.5 kcal/mol toward the straight conformation, providing direct (computational) support for the lattice model of microtubule assembly.

Specifically, at the earliest stages of tubulin assembly, the lattice model predicts that lateral contacts will be disadvantaged because the binding interface is disrupted in the bent conformation. These results suggest that even forming a single lateral pair is sufficient to largely shift the equilibrium toward the straight conformation, which then makes subsequent lateral additions more thermodynamically favorable.

#### Implications of conformational changes in tubulin on MT assembly

To begin to explore the functional consequences of an assembly-dependent conformational change in tubulin that underlies the lattice model, Rice et al. had used a simple two-step nucleation-elongation model to approximate the kinetics of the early stages of MT assembly. In keeping with experimental data, kinetic parameters were set to capture the low affinity of subunit addition during nucleation and a high affinity of subunit addition during elongation: the monomer concentration was set to 10 µM, the bimolecular on-rate constant to *k*_f_ = 1×10^6^ M^−1^s^−1^. Reverse rate constants for nucleation 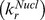 and elongation 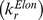 such were set such that their respective dissociation constants would be 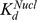 = 1 mM and 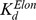 = 1 µM, respectively, and that the essence of the nucleation-elongation would be captured. The kinetic equations characterizing MT assembly of a nucleus size of *N* species (in which each species is represented by a heterodimer) have been described.^39^ Using these kinetic parameters and a nucleus size of *N* heterodimers, Rice et al. generated 500-second polymerization profiles for a range of straightening penalties from 0 to 1.6 *k*_B_T (∼1 kcal/mol). Using our data that the ensemble of bent states are ∼1 kcal/mol more energetically favorable than the straight conformer, Rice et al.'s model suggests that a ∼1 kcal/mol straightening penalty increases the time required to assemble into a 4-species nucleus by ∼10-fold. That a modest straightening penalty of 1 kcal/mol is sufficient to decrease the rate of MT assembly highlights the sensitivity of MT assembly kinetics to the initial stages of nucleation and elongation.

### Colchicine binding strongly shifts the population of soluble tubulin toward bent structures

#### Free energy profile of colchicine-bound tubulin

**Figure 5:**
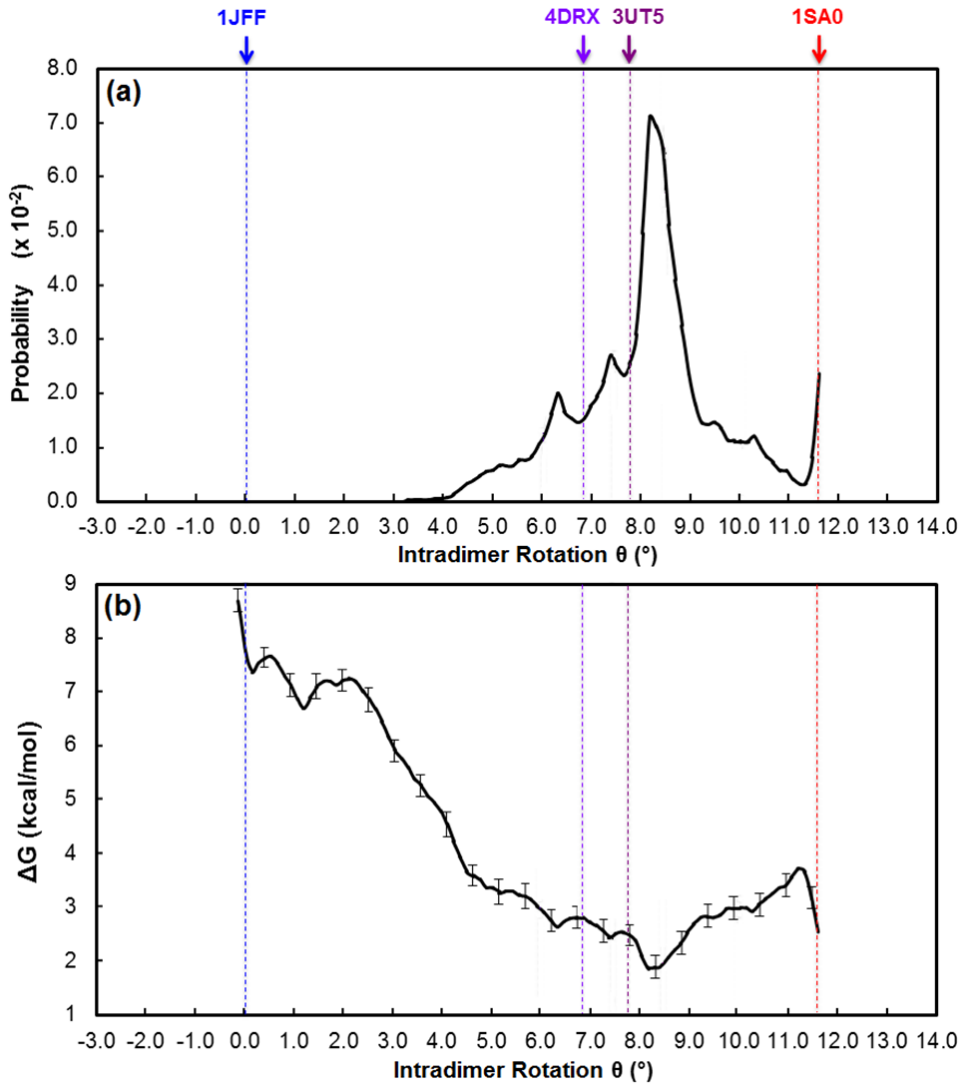
The (a) probability distribution and (b) free energy profile for the colchicine-bound tubulin as a function of intradimer rotation. Intradimer rotation of the endpoint structures, the “straight” taxol-liganded, zinc-stabilized protofilament tubulin and “bent” α_1_β_1_ heterodimer from the T2R-colchicine complex, are denoted at θ∼0° *(blue dash)* and θ∼12° *(red dash)*, respectively. Intradimer rotations observed in crystal structures of colchicine-bound tubulins are denoted as dotted lines at θ∼7.8° (both α_1_β_1_ and α_2_β_2_ heterodimers of T2R-colchicine-ustiloxin complex). Individual probability histograms corresponding to each umbrella window is shown in Figure S2b in Text S1.

Figure 5 shows the calculated free energy profile and probability distribution of the colchicine-bound tubulin complex as a function of the intradimer rotation. The estimated statistical error in the relative free energy profile ranges between 0.3–0.4 kcal/mol, confirming adequate umbrella sampling and overlap throughout all umbrella windows along the reaction coordinate, as shown in Figure S2b in Text S1. The restraints used in generating the PMF prevented colchicine from dissociating from tubulin. As expected, colchicine binding strongly disfavors the straight conformation, and the free energy decreases in a nearly linear manner, with no significant free energy barriers, as the intradimer rotation angle increases, up to the global free energy minimum observed at ∼9°. These results are consistent with previous findings that colchicine binds only to the bent conformer, thus inhibiting tubulin assembly into the microtubule lattice [7], [11], [20], [21]. The intradimer rotation angles observed in crystal structures of colchicine-bound tubulin range from ∼8–12° (Table 1), in good agreement with the PMF. It should be noted that the T2R-colchicine-ustiloxin structure is higher in resolution (2.7 Å) than the T2R-colchicine structure (3.6 Å), which we used to define the most highly bent end-point structure for the PMF. The two structures also have different space groups.

**Figure 6:**
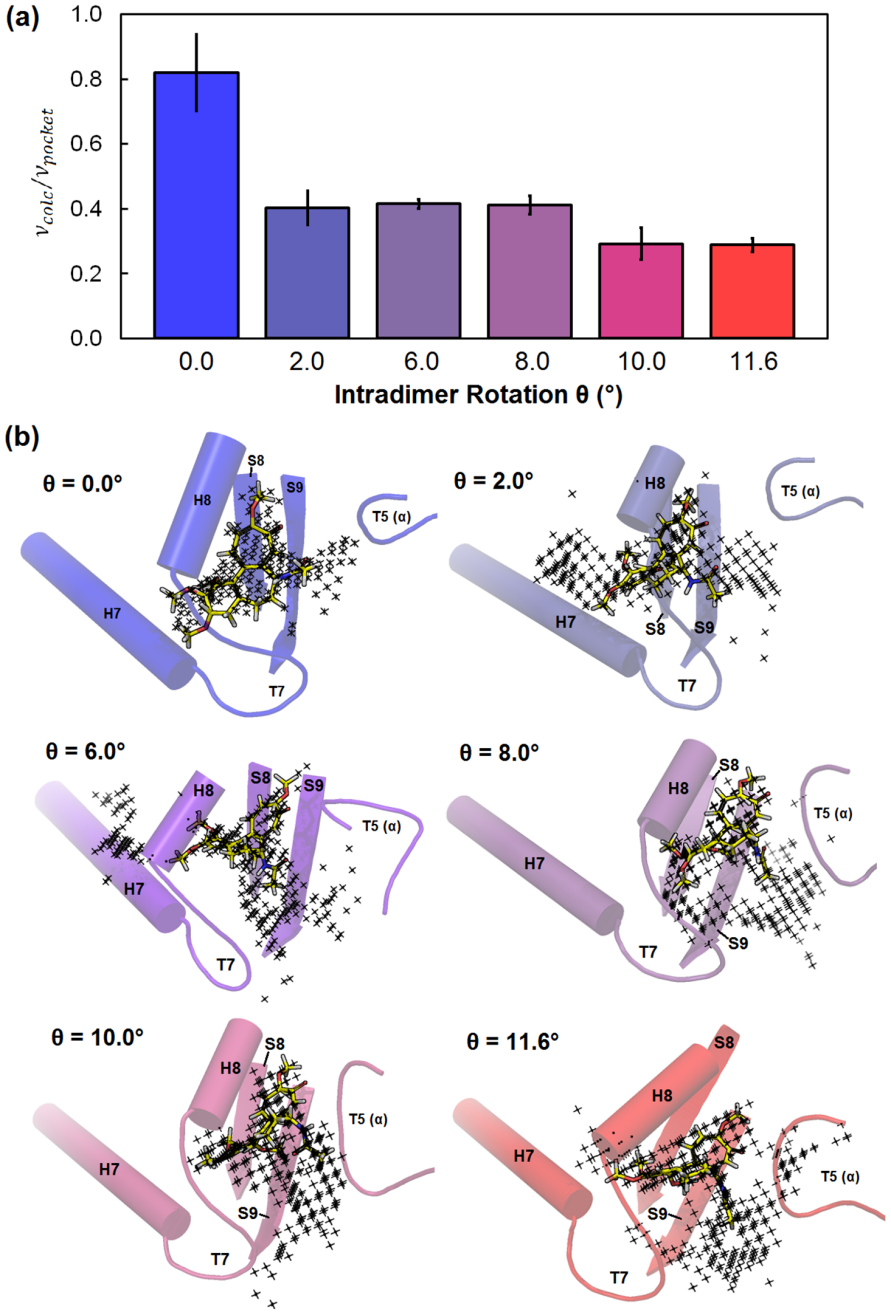
The colchicine binding pocket is sterically constrained in tubulin conformations with lower curvature. a) Ratios of the volume of colchicine with respect to the volume of the predicted binding pocket for representative colchicine-bound tubulin heterodimers with curvatures of θ∼0°, 2°, 6°, 8°, 10°, and 12°. b) SiteMap predictions of the colchicine binding site for colchicine-bound tubulin structures with intradimer curvature of θ = 0°, 2°, 6°, 8°, 10°, and 12°. For each colchicine-bound tubulin structure, the representative structure with the maximum SiteScore is displayed. Black dots represent the predicted binding pocket of colchicine. Structural elements comprising the colchicine-binding domain are rendered as cartoon.

Figure 6 depicts changes in the colchicine-binding pocket as a function of the tubulin intradimer rotation angle. One reason for colchicine strongly disfavoring the straight conformation is simply that the binding pocket becomes much smaller (235 Å^3^±29 Å^3^) than for fully bent tubulin (937 Å^3^±31 Å^3^ at θ = 12°). The pocket cannot collapse entirely in these simulations because colchicine is restrained to remain in the binding pocket. The colchicine-bound tubulin structures with ∼8° intradimer curvature also appear to be stabilized by hydrogen bonds with the backbone amide of α-Val 181 and side chain amine of β-Lys 350 (Figure S6 in Text S1), in agreement with previous research that Val 181 is essential for colchicine's biological activity [47].

The results presented here, predicting a continuum of intermediately bent tubulin states ranging from “straight” to “bent”, has potential implications for microtubule-directed drug discovery. In principle, it should be possible to identify compounds that can bind selectively to various “intermediately bent” states, as opposed to the strongly bent state stabilized by colchicine, or the straight state presumably stabilized by taxol. More broadly, our results suggest the potential utility of molecular dynamics methods (including more advanced methods like Markov State Models, which are not used here) to understanding how drugs or drug candidates modulate the free energy landscape of the target proteins. We expect this type of approach to be particularly relevant to understanding and ultimately designing allosteric modulators, and more broadly to how the chemical structure and binding mode of a drug dictate the ways in which it modulates the energy landscape.”

#### Conclusions

Structural and functional studies have previously demonstrated the existence of the straight-bent equilibrium for tubulin, and its relevance to microtubule assembly. Our new contribution here is to compute the free energy along a conformational coordinate connecting two limiting straight and bent structures. We had hypothesized that we would observe two free energy basins, corresponding to the straight and bent states, with a free energy barrier separating these, and that the bent state would correspond to the lowest (most favorable) free energy, consistent with the lattice model of microtubule assembly. The computed ‘potential of mean force’ along the chosen bending coordinate largely supports this hypothesis, but also suggests that the bent state is best understood as an ensemble of states with a significant range of intradimer rotation angles (4–13°). The global free energy minimum in the PMF lies at 6° intradimer rotation, whereas the colchicine-bound structure that we (and others) have used to define the ‘fully bent’ state lies at 12°, and is slightly higher in free energy. Our colchicine simulations also support the observation that colchicine biases towards conformations more bent than the 6° rotation angle calculated for free tubulin. Recent structures of tubulin co-crystallized with various small molecules and macromolecules support the prediction that tubulin can adopt a wide range of bent states.

Overall, the PMF predicts that the ensemble of bent states, taken as a whole, has a free energy that is lower than that of the straight state by about 1 kcal/mol, a modest value that is nonetheless sufficient to have a significant impact on polymerization kinetics, as represented (crudely) by a simple nucleation-elongation model. We emphasize that the precise value should not be over-interpreted, because the statistical uncertainty estimated for the PMF is ∼0.5 kcal/mol; in addition, the underlying molecular mechanics force field model also has limitations that could lead to systematic errors, the magnitude of which are difficult to estimate. An additional limitation is that the PMF only probes one linear coordinate connecting two structures that we chose as the extreme straight and bent states. There could exist other ‘off-pathway’ states, involving conformational changes orthogonal to and independent of the coordinate probed here, which could also have functional relevance. It is also highly likely that tubulin is capable of adopting bent conformations whose curvature is in the direction opposite of that of 1SA0, as explored by Voth and co-workers [13], and other intermediately bent tubulins in the continuum of structures predicted in our PMF. While unrestrained MD simulations do not have this limitation [12], [14], [18], it is extremely difficult from them to converge, i.e., to adequately sample the full conformational space. In future work, Markov State Models could potentially be used to more exhaustively characterize the free energy landscape, albeit with substantial computational expense [48].

Nonetheless, the *qualitative* predictions of the PMFs agree well with a variety of experimental observations, including the fact that the intradimer rotation and surface area buried between the α and β chains in experimental structures correspond well with low free energy portions of the PMF. We also demonstrate that tubulin binding to the drug colchicine, and binding to another tubulin in a lateral interaction, both *perturb* the PMF in ways consistent with expectations. Colchicine binding strongly biases the equilibrium toward the bent state, to the extent that the straight state is thermodynamically infeasible; we attribute this shift largely to simple steric considerations, where the binding pocket shrinks as tubulin straightens. Lateral tubulin binding shifts the conformational equilibrium in the opposite manner, such that the most extreme bent states become infeasible and the straight conformation corresponds to the global free energy minimum (although intermediately bent states may still be populated). This result is consistent with the lattice model of assembly, and additionally suggests that a single lateral interaction is sufficient to “pay” the free energy cost of straightening tubulin.

Our PMFs of unpolymerized tubulin and laterally-paired tubulins are based on tubulins bound with GDP at the β-subunit E-site. We had chosen to use GDP-bound tubulin because these were the only X-ray crystallographic structures available at the commencement of this study, and it would have been risky to artificially build in GTP. We have not considered in this work the role of nucleotide state at this site, a factor that has been considered in some prior MD simulations. Although SAXS experiments reveal that GTP- and GDP-bound tubulin adopt very similar, bent states in solution and that nucleotide choice does not influence colchicine binding, we cannot rule out the possibility that nucleotide state could modulate the PMF in some way, e.g., GTP shifting the equilibrium of the M-loop in a way that might additional favor lateral interactions. In principle, free energy simulations (PMFs or alchemical changes) could be used to address this issue in future work.

## Supporting Information

Text S1 **Supporting Information.** This section documents the supplementary figures and tables referenced throughout the text.
(DOCX)
