# Supplementary material for: The Free Energy Profile of Tubulin Straight-Bent Conformational Changes, with Implications for Microtubule Assembly and Drug Discovery
Source: PLoS Comput Biol. 2014 Feb 6;10(2):e1003464. doi: 10.1371/journal.pcbi.1003464 (PMC3916224; doi:10.1371/journal.pcbi.1003464)
Supplement: Text S1 — Supporting Information. This section documents the supplementary figures and tables referenced throughout the text. (DOCX) [file pcbi.1003464.s001.docx]

**Text S1. Supporting Information**

**
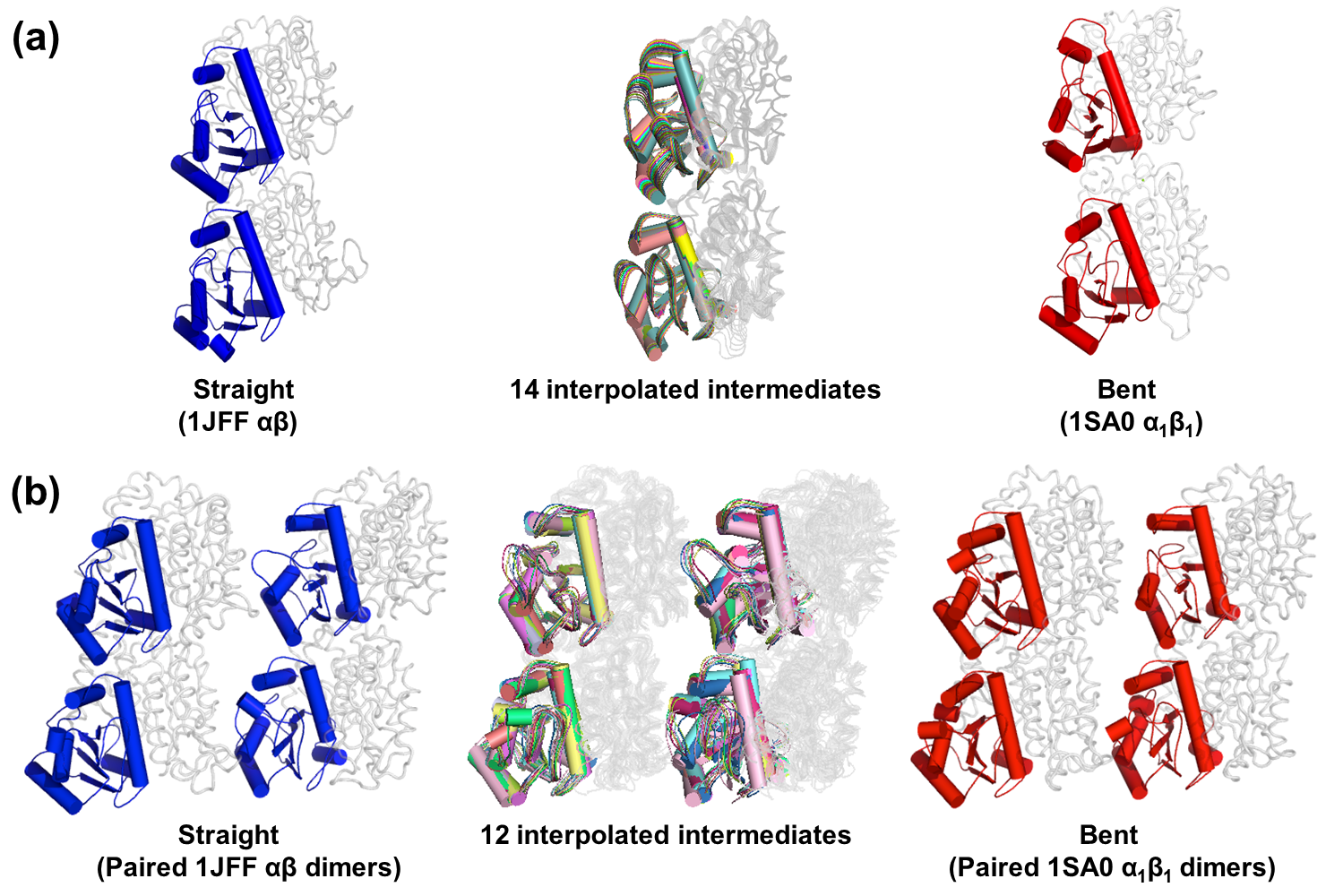
**

**Figure S1**. Equilibrated endpoint and interpolated intermediate tubulin structures obtained by means of linear interpolation using the Molmov morphing server for the a) unpolymerized tubulin and b) laterally-paired tubulins. Figure generated using PyMOL.

**
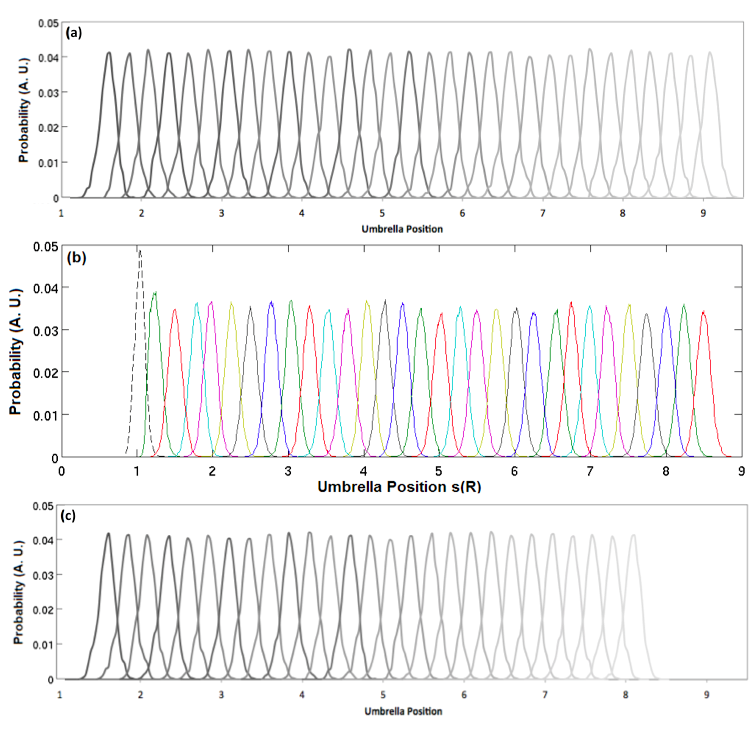
**

**Figure S2.** Probability histograms of the umbrella windows in the *s*(R) reaction coordinate of a) unpolymerized tubulin, b) colchicine-bound unpolymerized tubulin, and c) laterally-paired tubulins. For colchicine-bound unpolymerized tubulin, the dotted histogram represents the umbrella windows derived from a nodal structure manually extrapolated before *s*(R) = 1 by the average mean squared displacement of $\bar{\left[ R-R(i) \right]}\cong$ 0.65 Å, in addition to the endpoint and interpolated intermediate nodal structures.


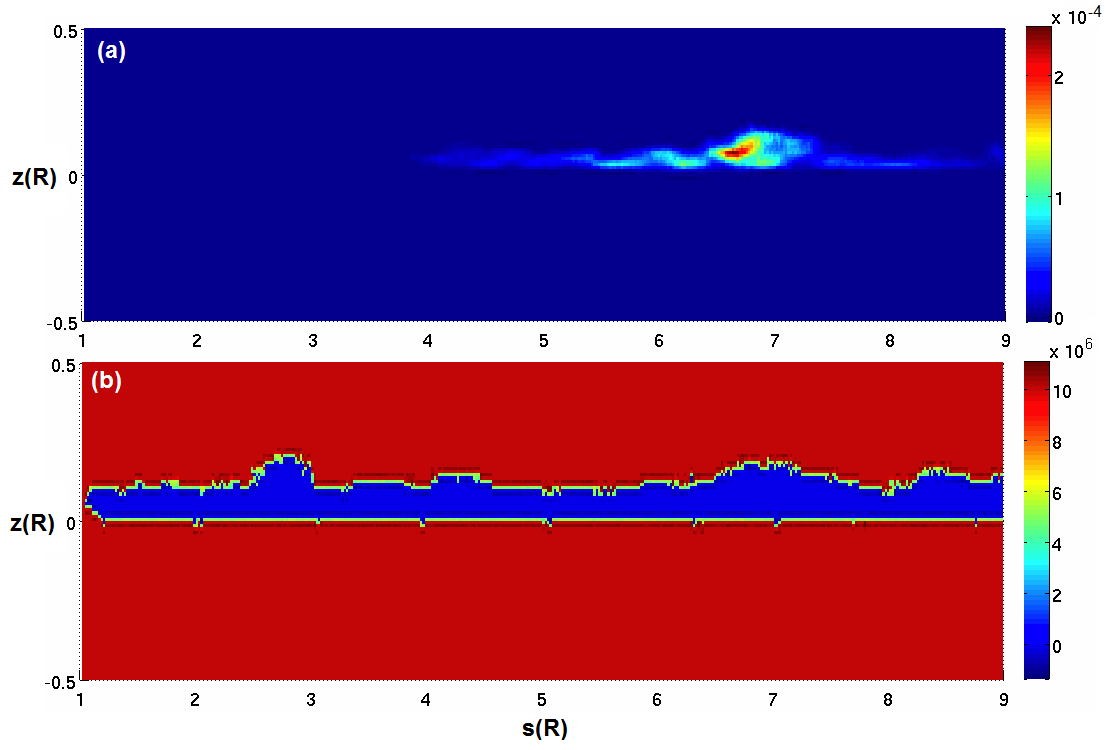


**Figure S3.** Multidimensional surface plots of the a) free energy space and b) probability distribution of colchicine-bound unpolymerized tubulin with respect to s(R) and z(R). Figure generated using MATLAB 7.0.


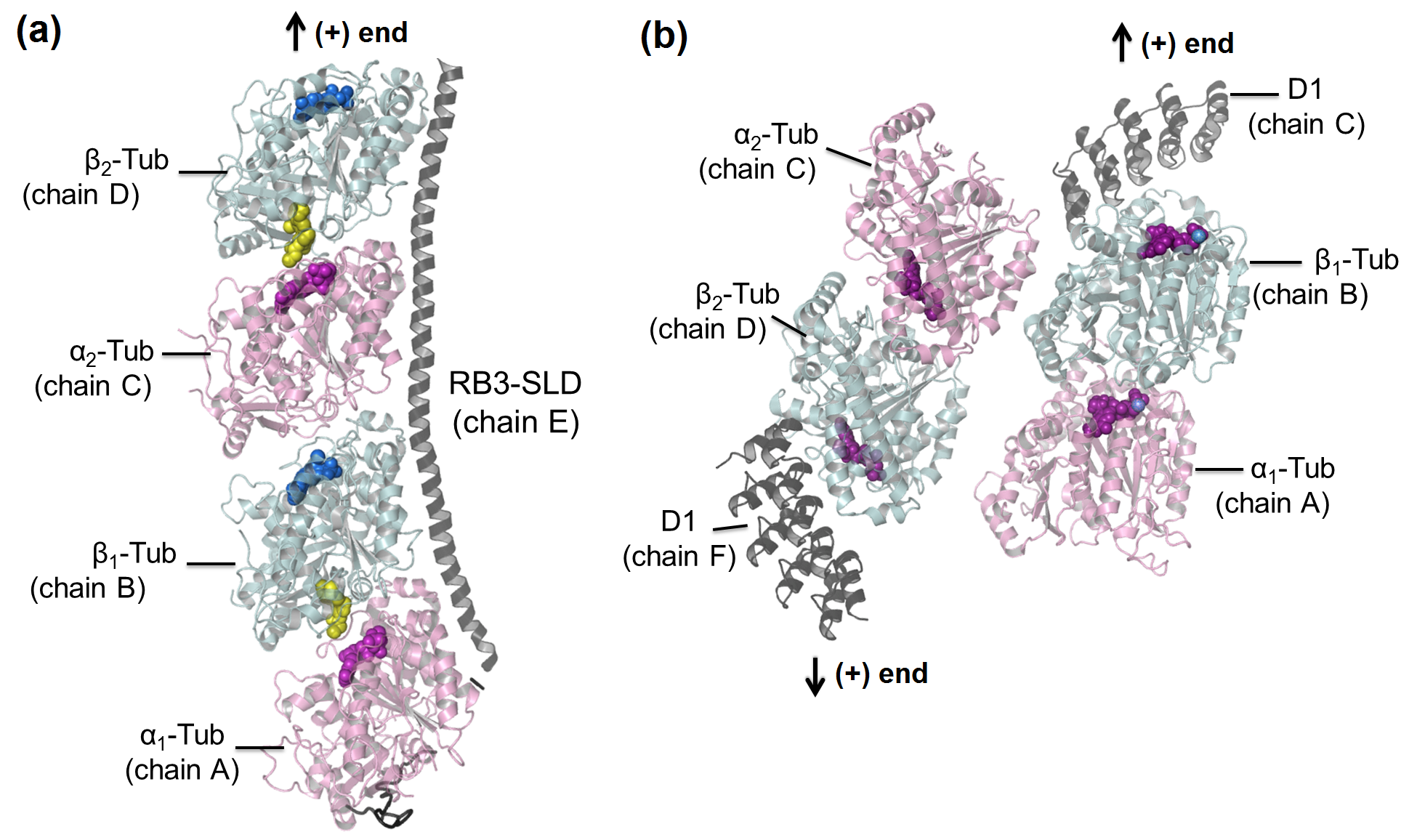


**Figure S4.** X-ray crystallographic structures of the T2R-colchicine and GTP-tubulin-D1 DARPin complexes. Shows the a) longitudinally-associated α_1_β_1_ and α_2_β_2_ heterodimers each bound with colchicine *(yellow spheres)*, together in a complex with RB3-SLD and b) α_1_β_1_ and α_2_β_2_ heterodimers each complexed with a D1 protein. GDP *(blue)* and GTP *(purple)* molecules are also rendered as spheres. Orientation of the β-subunit of each heterodimer towards the microtubule plus (+) end is denoted accordingly.

**Figure S5.** Comparison of measurement methods of tubulin curvature for fifteen representative structures along the reaction coordinate for unpolymerized tubulin in Figure 3. The Voth method for calculating the “intrinsic bending angle” is to measure the intersection angle of two least-square fitted-vectors, each defined through the center-of-masses of the N-terminal, intermediate, and C-terminal domains of each of the α- and β-subunits. The Jacobson method for calculating the “intradimer rotation” is to align the α-subunit H7 helix of the “bent” tubulin to the α-subunit H7 helix of the reference “straight” tubulin, followed by measuring the intersection angle of two least-square fitted-vectors, each defined through the center-of-masses of the α- and β-H7 helices, of the “bent” structure and “straight” structure. All angles for the Voth and Jacobson methods were calculated using the backbone N-Cα-C-O atoms. It is apparent that both methods capture the degree of tubulin curvature equitably.


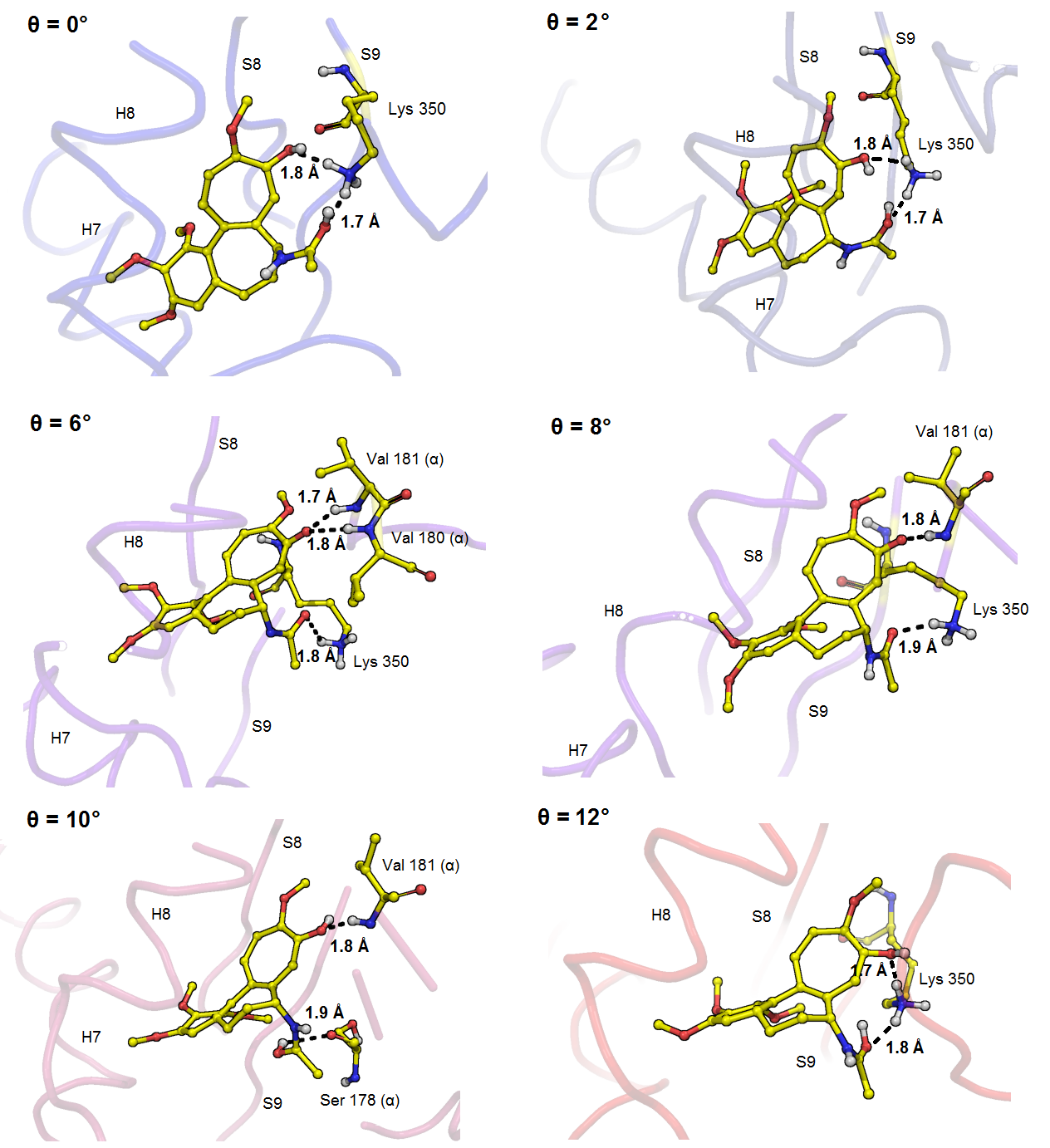


**Figure S6.** Hydrogen bonding between colchicine and tubulin in structures with varying intradimer curvature along the reaction coordinate for tubulin-colchicine complex.

| **Table S1. Intramonomer rotations of α- and β-subunits of interpolated tubulin structures along the conformational change coordinate.** Numbering of structures 1 to 15 reflects conformational change of the representative structures from the straight to bent endpoints. The angles were calculated in accordance with methods described in Knossow et al. *Nature* 2004 using backbone N-Cα-C-O atoms. | | |
| --- | --- | --- |
| **Structure No.** | **α-subunit Rotation (°)** | **β-subunit Rotation (°)** |
| 1* | 0.2* | 0.1* |
| 2 | 1.3 | 0.3 |
| 3 | 2.1 | 0.6 |
| 4 | 2.8 | 0.8 |
| 5 | 3.6 | 0.9 |
| 6 | 4.5 | 0.9 |
| 7 | 5.1 | 1.0 |
| 8 | 5.8 | 1.2 |
| 9 | 6.4 | 1.7 |
| 10 | 7.0 | 2.3 |
| 11 | 7.5 | 3.4 |
| 12 | 8.0 | 4.7 |
| 13 | 8.5 | 6.3 |
| 14 | 8.7 | 8.4 |
| 15 | 8.9 | 11.5 |

*Straight endpoint structure whose α- and β-subunit rotations are calculated with respect to those of the 1JFF structure.

| **Table S2a. Twist Angle α of tubulin heterodimers of laterally-paired tubulin structures along the conformational change coordinate.** Numbering of structures 1 to 13 reflects conformational change of the representative structures from the straight to bent endpoints. The twist angles were calculated in accordance with methods described in Grafmüller et al. *Structure* 2011 using backbone N-Cα-C-O atoms. | | |
| --- | --- | --- |
| **Structure No.** | **Twist Angle (°)**  **Left Heterodimer** | **Twist Angle (°)**  **Right Heterodimer** |
| 1 | 4.3 | 2.2 |
| 2 | 4.2 | 3.1 |
| 3 | 3.1 | 5.1 |
| 4 | 5.1 | 4.3 |
| 5 | 4.3 | 3.0 |
| 6 | 3.0 | 4.2 |
| 7 | 4.2 | 3.4 |
| 8 | 3.4 | 3.1 |
| 9 | 3.1 | 8.8 |
| 10 | 8.8 | 4.8 |
| 11 | 4.8 | 3.6 |
| 12 | 3.6 | 2.5 |
| 13 | 2.5 | 4.6 |

| **Table S2b. Twist angles of heterodimers of X-ray crystallographic structures of tubulin bound to** **RB3-SLD and D1-DARPin.** Enumeration of heterodimers α_1_β_1_ and α_2_β_2_ is in accordance with that shown in Figure S4 in Text S1. | | | |
| --- | --- | --- | --- |
| **Structure** | **PDB ID** | **Twist Angle (°)**  **α_1_β_1_** | **Twist Angle (°)**  **α_2_β_2_** |
| Taxol-bound tubulin* | 1JFF* | 1.4* | N/A |
| T2R-TTL-zampanolide | 4I4T | 6.8 | 5.6 |
| T2R-TTL-ADP | 4IHJ | 6.3 | 5.9 |
| T2R-TTL | 4I55 | 6.4 | 5.8 |
| T2R-TTL-epothilone A | 4I50 | 6.3 | 5.9 |
| T2R-colchicine | 1SA0 | 8.2 | 5.8 |
| T2R-podophyllotoxin | 1SA1 | 6.8 | 5.1 |
| T2R | 3RYC | 6.6 | 7.8 |
| GTP-tubulin-D1-DARPin | 4DRX | 7.7 | 8.3 |
| T2R | 3RYI | 6.7 | 8.5 |
| T2R | 3RYF | 6.3 | 7.6 |
| T2R-colchicine-ustiloxin | 3UT5 | 5.9 | 6.9 |
| T2R | 3RYH | 5.7 | 7.9 |
| T2R-vinblastine | 4EB6 | 5.1 | 7.5 |
| T2R | 3HKB | 8.6 | 6.5 |

*Control calculations of the “straight” tubulin were also performed.

| **Table S3. Residues of the H7 helices in the α- and β-subunits used in calculating the intradimer rotation angle.** | | | | |
| --- | --- | --- | --- | --- |
| **Structure** | **PDB ID** | **Heterodimer** | **α-H7 residues** | **β-H7 residues** |
| Docetaxel-tubulin* | 1TUB* | αβ | 224-243 | 222-242 |
| Epothilone A-tubulin* | 1TVK* | αβ | 224-242 | 222-242 |
| T2R-TTL-zampalonide | 4I4T | α_1_β_1_ | 223-244 | 223-244 |
|  |  | α_2_β_2_ | 224-243 | 224-243 |
| T2R-TTL-ADP | 4IHJ | α_1_β_1_ | 224-244 | 224-244 |
|  |  | α_2_β_2_ | 224-242 | 224-242 |
| T2R-TTL | 4I55 | α_1_β_1_ | 223-244 | 223-244 |
|  |  | α_2_β_2_ | 224-243 | 224-243 |
| T2R-TTL-epothilone A | 4I50 | α_1_β_1_ | 223-244 | 223-244 |
|  |  | α_2_β_2_ | 224-243 | 224-243 |
| T2R-colchicine | 1SA0 | α_1_β_1_ | 224-245 | 225-243 |
|  |  | α_2_β_2_ | 224-242 | 225-242 |
| T2R-podophyllotoxin | 1SA1 | α_1_β_1_ | 223-245 | 224-244 |
|  |  | α_2_β_2_ | 224-238 | 225-243 |
| T2R | 3RYC | α_1_β_1_ | 224-243 | 224-243 |
|  |  | α_2_β_2_ | 224-243 | 224-243 |
| GTP-tubulin-D1 DARPin | 4DRX | α_1_β_1_ | 224-243 | 224-243 |
|  |  | α_2_β_2_ | 224-243 | 224-243 |
| T2R | 3RYI | α_1_β_1_ | 224-243 | 224-243 |
|  |  | α_2_β_2_ | 224-243 | 224-243 |
| T2R | 3RYF | α_1_β_1_ | 223-244 | 223-244 |
|  |  | α_2_β_2_ | 224-243 | 224-243 |
| T2R-colchicine-ustiloxin | 3UT5 | α_1_β_1_ | 223-243 | 223-243 |
|  |  | α_2_β_2_ | 224-243 | 224-243 |
| T2R | 3RYH | α_1_β_1_ | 223-244 | 223-244 |
|  |  | α_2_β_2_ | 224-243 | 224-243 |
| T2R-vinblastine | 4EB6 | α_1_β_1_ | 223-243 | 224-238 |
|  |  | α_2_β_2_ | 224-239 | 224-239 |
| T2R | 3HKB | α_1_β_1_ | 224-244 | 224-242 |
|  |  | α_2_β_2_ | 224-245 | 225-242 |

*Control calculations of the “straight” tubulin were performed on the docetaxel- and epothilone A-bound, Zn^2+^-stabilized protofilament tubulin with respect to the taxol-bound, Zn^2+^-stabilized protofilament tubulin (α-H7: 224-242, β-H7: 224-243)
